# Harbingers of Disease: Decoding Shared Multi‐Omic Changes in Human and Mouse Models of Alzheimer’s Disease

**DOI:** 10.1002/alz.087010

**Published:** 2025-01-03

**Authors:** Vivek Swarup, Samuel Morabito, Emily Miyoshi, Elizabeth Head, Kim N. Green

**Affiliations:** ^1^ Institute for Memory Impairments and Neurological Disorders (MIND), Irvine, CA USA; ^2^ Mathematical, Computational and Systems Biology (MCSB) Program, UC Irvine, Irvine, CA USA; ^3^ University of California, Irvine, Irvine, CA USA; ^4^ The UC Irvine Institute for Memory Impairments and Neurological Disorders (UCI MIND), Irvine, CA USA

## Abstract

**Background:**

Alzheimer’s Disease (AD) presents a significant challenge in understanding its complex pathophysiology, owing to its multifaceted genetic and environmental factors. Despite extensive research, the translatability of findings from animal models to human conditions remains a critical hurdle. This study addresses the need to uncover shared molecular changes in AD by comparing human and mouse models, thereby enhancing our understanding of the disease’s underlying mechanisms and improving the prospects for effective treatments.

**Method:**

The study employed a comprehensive multi‐omic approach, integrating spatial transcriptomics (ST) and single‐nucleus RNA‐sequencing (snRNA‐seq) data from postmortem human prefrontal cortical tissue and 5xFAD mouse brains across various stages of AD pathology, including AD in Down Syndrome (DS). An unbiased clustering algorithm, BayesSpace, was used to identify distinct clusters in both human and mouse datasets. Cross‐species differential expression analysis and multi‐scale co‐expression network analysis using hdWGCNA were conducted. These analyses were complemented with fluorescent amyloid imaging to correlate gene expression changes with amyloid pathology.

**Result:**

The analysis revealed evolutionary‐conserved AD transcriptional changes, particularly in genes associated with amyloid‐beta response and microglial activation. The study identified upregulated genes with age in the mouse model, with variances across different brain regions. Co‐expression network analysis exposed amyloid‐associated gene modules that were consistent across species. Additionally, the integration of spatial and single‐cell data elucidated cellular diversity and transcriptomic alterations within the AD brain, providing a spatial context to the observed molecular changes.

**Conclusion:**

This study successfully decodes shared multi‐omic changes in human and mouse models of Alzheimer’s Disease, highlighting key molecular similarities and differences across species. The findings underscore the significance of a multi‐faceted, integrated omic approach in understanding AD pathogenesis. This cross‐species analysis not only advances our knowledge of the molecular underpinnings of AD but also opens avenues for developing more accurate and translatable AD models, ultimately guiding the discovery of potential therapeutic targets.

